# Epigenetic modulation of fungal pathogens: a focus on *Magnaporthe oryzae*

**DOI:** 10.3389/fmicb.2024.1463987

**Published:** 2024-10-28

**Authors:** Hafiz Muhammad Usman Aslam, Mohamad Chikh-Ali, Xin-Gen Zhou, Shouan Zhang, Steven Harris, Ashok K. Chanda, Hasan Riaz, Akhtar Hameed, Saba Aslam, Nabil Killiny

**Affiliations:** ^1^Department of Plant Pathology, San Luis Valley Research Center, Colorado State University, Fort Collins, CO, United States; ^2^Department of Plant Pathology, Institute of Plant Protection, MNS-University of Agriculture, Multan, Pakistan; ^3^Texas A&M AgriLife Research Center, Beaumont, TX, United States; ^4^Department of Plant Pathology, Tropical Research and Education Center, University of Florida, IFAS, Homestead, FL, United States; ^5^Department of Plant Pathology, Entomology and Microbiology, Iowa State University, Ames, IA, United States; ^6^Department of Plant Pathology and Northwest Research and Outreach Center, University of Minnesota, St. Paul, Crookston, MN, United States; ^7^Department of Plant Pathology, University of Agriculture, Faisalabad, Pakistan; ^8^Department of Plant Pathology, Citrus Research and Education Center, University of Florida, IFAS, Lake Alfred, FL, United States

**Keywords:** genomics, strain improvement, filamentous fungi, extracellular enzymes, pathogen-induced epigenetic changes

## Abstract

Epigenetics has emerged as a potent field of study for understanding the factors influencing the effectiveness of human disease treatments and for identifying alternations induced by pathogens in host plants. However, there has been a paucity of research on the epigenetic control of the proliferation and pathogenicity of fungal plant pathogens. Fungal plant pathogens such as *Magnaporthe oryzae*, a significant threat to global rice production, provide an important model for exploring how epigenetic mechanisms govern fungal proliferation and virulence. In *M. oryzae*, epigenetic alterations, such as DNA methylation, histone modification, and non-coding RNAs, regulate gene expression patterns that influence the pathogen’s ability to infect its host. These modifications can enhance fungal adaptability, allowing the pathogen to survive in diverse environments and evade host immune responses. Our primary objective is to provide a comprehensive review of the existing epigenetic research on *M. oryzae* and shed light on how these changes influence the pathogen’s lifecycle, its ability to invade host tissues, and the overall severity of the disease. We begin by examining the epigenetic alterations occurring in *M. oryzae* and their contributions to the virulence and proliferation of the fungus. To advance our understanding of epigenetic mechanisms in *M. oryzae* and similar plant diseases, we emphasize the need to address unanswered questions and explore future research directions. This information is crucial for developing new antifungal treatments that target epigenetic pathways, which could lead to improved disease management.

## Why do we study epigenetics in Fungi?

The cell has the ability to modify gene expression in response to its internal conditions and external influences. Remarkably, these alterations in gene expression can persist even after the initial stimulus has been removed, leading to the development of cell clones that maintain the modified behavior. This phenomenon is known as epigenetics, wherein most cases of heritable shifts in cell state do not involve alterations to the DNA sequence itself. In humans, epigenetic mechanisms play crucial roles in determining cell types, silencing repetitive DNA sequences, regulating the parent-of-origin expression of monoallelic genes, and causing abnormal gene expression in conditions of cancer.

These processes are governed by various fundamental mechanisms, including positive feedback loops regulated by transcription factors, DNA methylation, and modifications to histone proteins. The majority of research in this field has been done using mammalian models, but many interesting mechanisms have also been discovered in other branches of the evolutionary tree. This article focuses on the unique epigenetic innovations found in fungi, serving as an example of evolutionary novelty. Among these discoveries are some that initially appear astonishing, such as chromatin prions and the erasure of centromeric nucleosomes leading to kinetochore inactivation. On the other hand, certain findings, like the Darwinian development of DNA methylation patterns over millions of years timescales, seem more plausible.

Fungi are an intriguing subject of study for a variety of reasons, including their suitability for experimentation, which simplifies the testing of hypotheses. Brewer’s yeast, *Saccharomyces cerevisiae*, has been studied extensively and has provided crucial insights into the biology of eukaryotic cells. However, it is becoming clear that partly due to ancestral evolutionary loss events, not all highly conserved elements of eukaryotic cellular behavior can be replicated in a single species. Valuable insights and unexpected revelations have emerged from in-depth studies of a small subset of other fungal species. For instance, it has been observed that mutations in the RNAi machinery of *Schizosaccharomyces pombe* can affect repressive histone H3 lysine 9 methylation (Cutter Dipiazza et al., 2021). Epigenetic mechanisms play a crucial role in plant interactions with fungi, significantly influencing processes like root nodule symbiosis and mycorrhizal associations (Martin et al., 2017; Zanetti et al., 2024). Plant pathogenic fungi, responsible for a large portion of plant diseases that jeopardize global food security, exhibit remarkable versatility in colonizing host cells while evading plant immune responses. The role of epigenetics in this context has been highlighted through the ability of plant pathogenic fungi to modify effector protein genes into avirulence (Avr) factors. Numerous studies have documented the epigenetic regulation of these effector protein genes (Gijzen et al., 2014), emphasizing the control of virulence and avirulence factors by phytopathogenic fungi (Qutob et al., 2013), including pathogens such as *Phytophthora sojae* (Na et al., 2014), *Ustilago hordei* (Ali et al., 2014), and *Magnaporthe oryzae* (Xu et al., 2022a, 2022b).

However, the fungal kingdom is vast, comprising over a million different species, and our exploration of this ancient microbial civilization has only scratched the surface of its mysteries. High-throughput DNA sequencing has made it possible to obtain the genome sequences of fungi, but gaining a deep understanding of their biology will require experimental examinations. This, in turn, necessitates scientific communities that are willing to invest their efforts in developing tools and conducting comprehensive explorations.

### Introduction

Epigenetics is generally defined as the study of heritable variations in gene function during mitosis and/or meiosis, excluding mutations in the DNA sequence. It is the study that goes beyond Mendelian principles (Madhani, 2021). As soon as ‘epigenetics’ was first introduced by Conrad Waddington in the early 1940s, research into the topic exploded. Initially, it referred to how phenotypes result from genotypes during development. Presently, epigenetics pertains to the molecular mechanisms influencing genome function regardless of sequence and their stability through cell division. While DNA mutations drive evolution and phenotypic diversity, they do not fully explain the breadth of phenotypic expression (Aghcheh and Kubicek, 2015). Epigenetics provides an intriguing explanation for the observed disparity between DNA mutations and phenotypic changes, attributing phenotypic variation to the gain of the loss or retention of epigenetic alterations across generations. Moreover, epigenetics, in combination with genetic diversity, may alter the foundation of inheritance under environmental influences (Skinner, 2015).

Epigenetic mechanisms have been extensively studied to determine their role in a wide range of biological methods, including cell variation and senescence, transposon silencing, embryonic development, plant growth, plasticity of neuronal cells, phenotypic variations between monozygotic twins, eye color variation in *Drosophila melanogaster*, autoimmune diseases, oncogenesis, obesity, and behavioral ailments like depression and schizophrenia (Thapa and Shrestha, 2020). Epigenetic regulation is considered a ubiquitous mechanism for modulating gene expression, despite our limited understanding of it.

The widespread presence, extensive variation, and adaptable metabolism of filamentous fungi make them crucial microorganisms for contemporary biotechnology. Since the era of antibiotic discovery, fungi have been viewed not just as potential pathogens but also as valuable sources of microbial cells, enzymes, compounds, and medications (Sharma Ghimire and Jin, 2017). For instance, the market for plant biomass-degrading enzymes produced by filamentous fungi, currently valued at €4.7 billion, is predicted to double in the next decade (Meyer et al., 2016).

Epigenetics is employed to explore alterations induced by pathogens in host plants that impact their immunity. Non-pathogenic models like *Saccharomyces cerevisiae* (budding yeast), *Schizosaccharomyces pombe* (fission yeast), and *Neurospora crassa* (filamentous fungus) have all played significant roles in the extensive research on epigenetic regulation in fungi (Malagnac and Silar, 2011; Kronholm et al., 2016; Poças-Fonseca et al., 2020). These studies have found epigenetic mechanisms such as DNA methylation, histone changes, and RNA silencing. Surprisingly, different epigenetic regulatory methods have been detected either as nonexistent or present in fungal models. Some RNAi-based silencing approaches, such as quelling and meiotic silencing by unpaired DNA (MSUD), have been known in *N. crassa* but not in *S. cerevisiae* (Hammond et al., 2013). Similarly, despite substantial research into the involvement of RNAi in heterochromatin formation in *S. pombe* (Thon et al., 2005), no such evidence was discovered in *N. crassa*. The diverse array of epigenetic processes observed in the phytopathogenic fungi serves as a fascinating illustration of the intricate nature of epigenetic observation, which warrants further study to uncover analogous, alternate, or unique regulatory paths. Recent advances in the exploration of epigenetics in non-phytopathogenic fungi have complicated our understanding of how epigenetics impacts the control of plant-fungal virulency. The acquisition of further information regarding the epigenetics of fungal pathogenesis is of extreme importance because of the evident research gap that exists in our conception of fungal pathogenesis, mainly concerning the discrepancies between phytopathogenic and non-pathogenic models.

In view of the above-mentioned literature gap, we intend to study the *M. oryzae*, the responsible cause of rice blast, as a model system for identifying the epigenetic processes governing the pathogenesis and development of the fungus. *M. oryzae* causes global economic losses, posing a severe threat to food security, with projected losses of up to 30% of the entire rice harvest (Martin-Urdiroz et al., 2016; Aslam et al., 2019). In addition, the fungal disease affects a wide variety of host plants (wheat, rice, oat, barley, millet etc.), causing major issues in many regions (Anwaar et al., 2020; Aslam et al., 2022). The alarming scale of *M. oryzae* damage is certainly reason enough to investigate this pathogen, but the fact that we already have complete genomes for both *M. oryzae,* and its rice host makes this a particularly amenable experimental model for learning about plant-fungus interactions. Numerous studies have provided evidence of the significance of epigenetics in facilitating the long-term survival of plant pathogens across diverse host environments (Castonguay and Angers, 2012). Despite being understudied, epigenetic research has begun to shed light on key questions concerning the pathogenicity and other infection-related processes of *M. oryzae.*

Considering the importance of *M. oryzae* and the prospects of epigenetics, understanding the plant pathogenicity associated with *M. oryzae* could provide insights into (i) how epigenetics regulates the development of disease transmission in other associated fungal phytopathogens, (ii) finding key epigenetic alterations as infection indicators for various fungal diseases, (iii) advancing our understanding of host-pathogen interactions using genomic and testing tractability; and (iv) facilitating the development of preventive and regulatory measures against fungal diseases.

In this review, we use *M. oryzae* as a model to highlight the importance of epigenetics in regulating fungal development and pathogenicity and to elaborate on some of the details that have yet to be uncovered. Therefore, we begin discussing the predominant epigenetic processes in *M. oryzae*. Some of these mechanisms, including quelling, sRNA-directed heterochromatin formation, and meiotic silencing due to unpaired DNA, have not been previously documented in *M. oryzae* and other fungal infections. The purpose of these brief explanations of as-yet-undiscoverable mechanisms is to stimulate future research into the possibility of discovering more, comparable, or different mechanisms of epigenetic control. In addition, we highlight some important, as-yet-uninvestigated regions of epigenetic control that may shed light on the factors that contribute to the survival, dissemination, and expansion of *M. oryzae* and other pathogenic fungi. Ultimately, we draw attention to the obvious deficiency in exploring the epigenetics of fungal infections in crops and emphasize the epigenetic alterations that can be employed as targets in the fight against fungal plant diseases.

## Epigenetic mechanisms in fungi

Epigenetics means cellular modifications that occur independently of changes in the DNA sequence. These modifications involve temporarily influencing the expression of specific genes without altering DNA sequences or the coding of proteins. There are two major types of epigenetic modifications: RNA-based and chromatin-based modifications.

The significance of uncovering epigenetic patterns in fungi has substantial implications for survival, virulence, and secondary metabolism. The epigenetic regulation in phytopathogenic fungi enables them to counter plant defenses evading host immunity (Gijzen et al., 2014). The identification of epigenetic switches in plant pathogenic fungi greatly enhances our understanding and ability to modify host plant genetic and biochemical mechanisms by regulating gene expression, chromatin remodeling, RNAi-based interventions, and changes in secondary metabolites pathways (Mierziak and Wojtasik, 2024; Zhang et al., 2024).

### RNA-based modifications

The RNA-based modifications encompass processes such as non-coding RNAs and RNA interference (RNAi). RNAi involves a fundamental pathway that includes the action of endonuclease Dicer and RNA-dependent RNA polymerases. Together, they generate small interfering RNAs (siRNAs) that induce RNA interference (RNAi). Processed siRNAs are incorporated into the Argonaute complex, enabling the targeted binding to complementary RNAs, leading to their degradation or inhibition of translation. Additionally, the RNAi machinery can sometimes repress gene expression by recruiting heterochromatin proteins to bind to the target genes (Grewal and Elgin, 2007).

In contrast to tiny nuclear RNAs, long non-coding RNAs (lncRNAs) are RNA molecules that are typically greater than 200 bp. Moreover, RNA polymerase II is responsible for transcribing lncRNAs, while RNA polymerase III handles the transcription of most small nuclear RNAs. Similar to mRNAs, many lncRNAs go through processes such as splicing, polyadenylation, and 5′ capping. Interestingly, the proportion of lncRNAs in the human genome compared to gene-coding RNAs is almost four times higher (Rao, 2017). Exosomes are responsible for degrading lncRNAs, which are mostly localized in the nucleus. As a result, they are initially disregarded as random transcriptional phenomena. Despite this, a number of research among others, have found evidence that long noncoding RNAs are involved in epigenetic gene regulation (Morlando et al., 2014).

### Chromatin modifications

The chromatin modifications resulting from chemical and structural changes have been extensively studied (Kramer et al., 2023). Chemical alterations comprise histone post-translational modifications (PTMs) and DNA methylation, while structural alterations involve chromatin transformation and DNA–DNA interactions. Many PTMs can occur on the N-terminal tail of histones in nucleosomes, such as phosphorylation, ubiquitination, and sumoylation while others are alkylation (Sadakierska-Chudy and Filip, 2015). These modifications alter the structure of chromatin, making some areas of the genome more accessible to transcription factors while limiting their ability to bind to transcription machinery in other regions. Below are some examples of epigenetic alterations.

**Figure 1:**
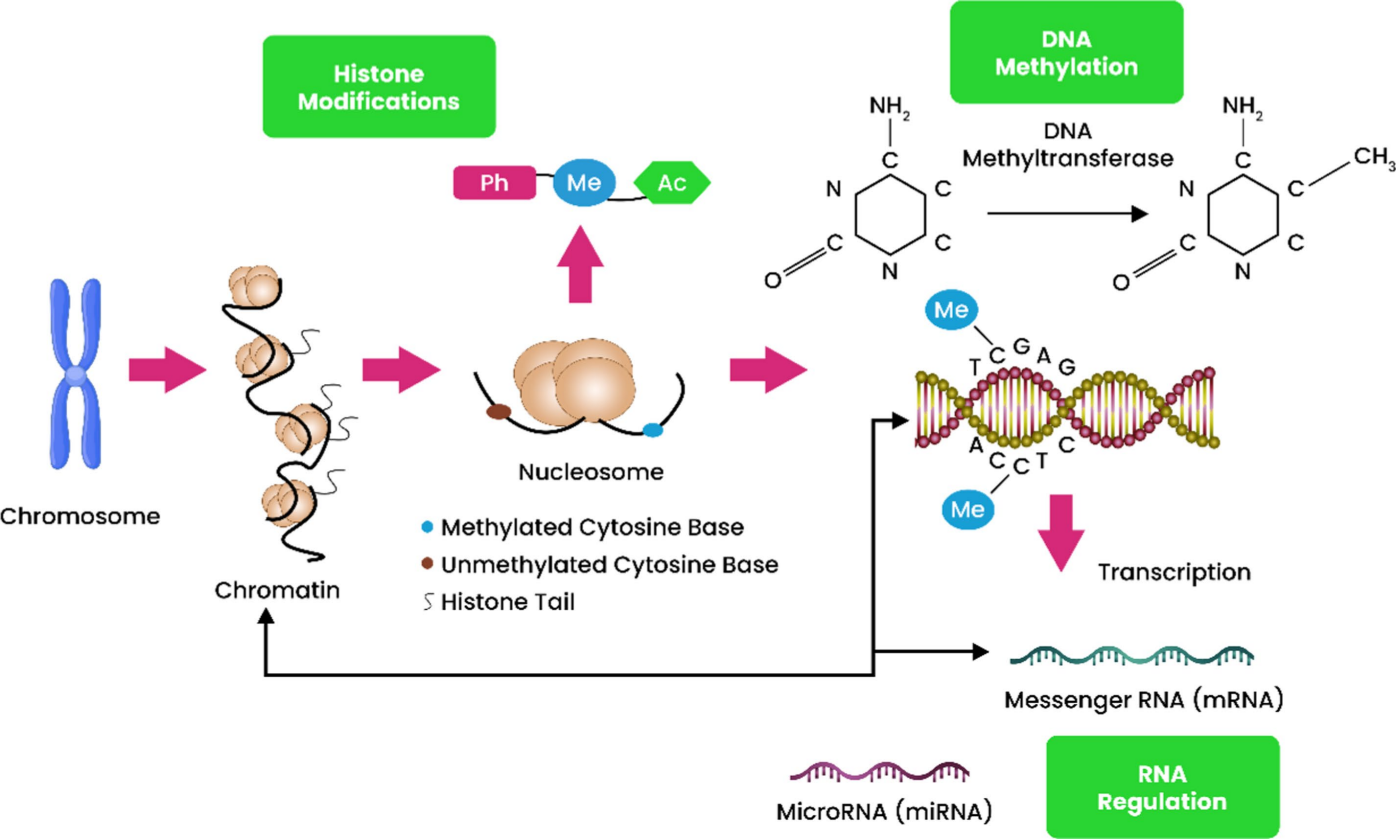
DNA methylation, histone posttranslational modifications (HPTMs), and microRNA regulation include epigenetic processes. HPTMs and DNA methylation govern pre-transcription initiation gene expression. Both mechanisms restructure the chromatin framework, making genes more or less responsive to transcription factors depending on epigenetic changes. In contrast to DNA methylation and HPTMs, miRNAs regulate gene expression post-transcriptionally. By binding to mRNAs, miRNAs negatively regulate genes by destroying or repressing translation.

- DNA methylation: The DNA base modification 5mC is created when a methyl group is added to a cytosine base, as described by Chen et al. (2017). This change can also be found in the genome of certain fungi, in addition to being common in the human genome. Methylation of adenine bases is a process that is seen in some organisms and can play an important role in their biology (Figure 1) (Kumar et al., 2018).
- Histone modifications: Histones make up the bulk of nucleosomes and serve as substrates for a number of PTMs (Zhao and Garcia, 2015). Methylation, acetylation, and phosphorylation are three PTMs that have been extensively studied and among them, few of these shifts are fixed, while the vast majority are merely in flux. Furthermore, steric restrictions may prevent the addition of additional PTMs to the same or neighboring residues if one PTM is already present. Protein arginine methyltransferases (PRMT), histone methyltransferases (HMT), and lysine acetyltransferases (KAT), are enzymes involved in mediating these alterations, while lysine demethylases (KDM) and lysine deacetylases (KDAC) are enzymes involved in removing them (Itoh, 2020). Histone-modifying known as KDACs and KATs are commonly denoted by their respective acronyms, HATs and HDACs. The accessibility of histone modifications to transcription factors, enhancers, and chromatin remodeling proteins can regulate gene expression. Acetylated histones, for instance, cause the chromatin to adopt an open conformation that facilitates transcription, while deacetylated histones cause the chromatin to adopt a compacted state that blocks transcription. Transcription is dynamically regulated by HDACs and HATs because they catalyze reversible processes (Clapier and Cairns, 2009).
- Chromatin transformation: In contrast to earlier concepts that categorized chromatin as either euchromatin (transcribed) or heterochromatin (non-transcribed), chromatin structure exhibits a remarkable degree of dynamism. What is crucial for transcription initiation is the chromatin’s capacity to undergo dynamic alterations in nucleosome positioning across the genome. To achieve chromatin remodeling, ATP-dependent nucleosome remodelers are indispensable. Two extensively studied examples of chromatin remodelers are ISWI and SWI/SNF (Erkina et al., 2010). Another significant factor impacting transcription and influencing chromatin compaction or relaxation is the process in which DNA sequences form loops, creating three-dimensional connections. The relationship between enhancers and promoters is a well-researched instance of this kind of interaction, as it facilitates the recruitment of transcription factors, initiating the transcription process. It underlines the complex nature of chromatin and its ability to regulate gene expression (Popay and Dixon, 2022).

The chromatin or RNA-based epigenetic changes provide an additional channel to react in response to environmental changes. The epigenetic alterations are transient and reversible along with a transgenerational effect which enables it to directly change the DNA sequence of an organism. Fungi are versatile and have the ability to withstand environmental stresses, such as drug exposure, by increasing their phenotypic plasticity through epigenetic modifications (Turner, 2009).

### Transcriptional regulation in *M. oryzae*

The epigenetic phenomenon includes DNA methylation, sRNA, and histone modifications which regulate the genes in eukaryotes (Simon and Meyers, 2011). The aforementioned epigenetic mechanisms are employed by fungi in the development of their biology including pathogenicity. In this paper, we have outlined major epigenetic means responsible for the virulence evolution in *M. oryzae* and its similarities with other fungal diseases of plants. We have also documented the role of epigenetic mechanisms in controlling fungal plant diseases, particularly rice blasts. Notably, three types of epigenetic changes *viz.* DNA methylation, histone protein alterations, and short RNAs (sRNAs) have been documented in *M. oryzae* and other fungal pathogens.

### DNA methylation

DNA methylation, a chemical alteration of DNA used to control gene expression, is an epigenetic procedure. It regulates gene expression by shifting the specificity of DNA-binding proteins or by enlisting proteins with roles in gene silencing. DNA methylation is hypothesized to be involved in a wide variety of cellular and hereditary processes (Reik and Lewis, 2005), including, but not limited to, X chromosome inactivation, genetic imprinting, gene silencing, and cell differentiation. The gene promoter regions are extensively studied in eukaryotes for gene activation and suppression which is associated with hypomethylation and hypermethylation, respectively. The DNA methyltransferases (DNMTs) are a class of enzymes, tasked with the incorporation of a methyl group to the 5′ carbon of cytosine. The transcription machinery is challenged by the methylation of 5′ regulatory regions of the genes by limiting its access to the genes (Uysal et al., 2015). DNA methylation involves induction or suppression of the transcription process through histone modifications followed by compaction or relaxation of chromatin assembly. Shen et al. (2014) studied the complex nature of epigenetically induced gene regulation. The study also revealed the dynamic role of RNA methylation and demethylation in mRNA production and movement.

*M. oryzae*, like other eukaryotes, undergoes cytosine methylation (C5-methyl-cytosine, 5mC), in contrast to the adenine methylation (N6-methyladenine, 6 mA) seen in prokaryotes. DNA methyltransferases also referred to as DNMTs, are an assembly of specialist enzymes liable for facilitating the methylation of cytosine bases inside DNA molecules (Jones and Liang, 2009). Two putative DNMTs*, MoDIM-2* and *MoRID*, were identified by BLAST searches (Poças-Fonseca et al., 2020). Alterations in vegetative development were observed in mutant strains of the relevant genes. Furthermore, through RNA-seq analyses, it was observed that there are location-specific differences in the transcript levels of transposable elements between the wild-type and the DModim-2 strains. While earlier studies with loss-of-function mutations in a different DNMT gene (*MoDMT1*) from various isolates had suggested a different outcome (Ikeda et al., 2013), these new findings underscore the critical role of DNA methylation in the life cycle and genome protection of *M. oryzae.*

DNA methylation is known to play an essential role in determining the extent of pathogenicity in *M. oryzae*. *Magnaporthe* Ty3-retrotransposon happens to be a long terminal repeat whose DNA methylation level differs among various *M. grisea* subgroups (Ikeda et al., 2001; Chadha, 2021). Several DNA methylation positions were observed through whole genome analysis of *M. oryzae* during different development stages. Mycelial DNA along with isolated DNA from conidia and appressoria were subjected to bisulfide sequencing for the identification of multiple methylation sites. Normally, the methylation of individual mC sites is between 20 and 30%. The existence of transposable element (TE)-enriched and TE-poor sections inside fungal mycelia exhibited different patterns of substantial methylation, revealing a unique targeting of methylation for TE regions. Apart from methylation in the mycelial stage, the frequency of methylation in conidia and appressoria is relatively low (Li et al., 2017). The scientific observation has determined the DNA methylation pattern in pathogens which tends to be dynamic and dependent on the type of fungal isolates or strains, the developmental stage of fungi, and growth conditions (He et al., 2020). To understand the evolution of DNA methylation, concerning fungal virulence, a comprehensive study may help explore the fungal methylome of several isolates under different growth conditions. Moreover, future discoveries in fungal epigenetics and DNA methylation will deepen our understanding of fungal biology, particularly regarding adaptability and virulence. Advances in high-resolution methylation profiling will illuminate dynamic patterns across different species and habitats, ultimately guiding strategies for disease control.

### Relics of repeat-induced point (RIP) mutation

*Neurospora crassa*, a model organism for studying fungal epigenetics, exhibits the intriguing phenomenon known as repeat-induced point (RIP) mutation. This mechanism enhances genomic stability and evolution by altering the genome in response to repetitive DNA sequences. RIP primarily targets duplicated genes and transposable elements, occurring when organisms encounter these repeated sequences during sexual reproduction. The association between RIP mutation and DNA methylation, a method imperative for gene silencing with the homology-based genome defense system, has been established (Chang et al., 2019). The fungal mating stage, or haploid phase, is a leading part of the growth cycle, transition mutations, which alter G:C to A:T, are added to repeat or duplicate sequences in the genome. This occurs regardless of whether the repeats or duplicates originate from internal or external sources. Repeatedly Interacted Protein (RIP) has been demonstrated to influence DNA methylation in the genome, though this effect is indirect.

Genome-wide investigation of the fungus *N. crassa* shows that the majority of the methylated areas are vestiges of transposons that are inactivated via RIP (Freitag et al., 2004). Nakayashiki et al. (2001) showed that a RIP-like process silenced the *M. grisea* MAGGY transposons. Traditionally, RIP predominantly occurs at 5’-C-phosphate-guanine (3’-CpG) dinucleotides, and sequences subjected to RIP are frequently linked with cytosine methylation in sexually reproducing fungi. However, there are laboratory reports of *M. grisea* in the sexual stage. Therefore, MAGGY retrotransposons and hygromycin B phosphotransferase were introduced into *M. grisea* and then crossed to test for the presence of a RIP-like mechanism in this fungus. Sequence analysis of the F1 offspring of *M. grisea* showed regular transition changes (A/Tp) and Cp (A/T) following crossing, indicating the presence of RIP-like mechanisms. Six *M. grisea* samples were examined, and it was shown that the newly discovered Ty3 retrotransposon, Pyret, has RIP-like transitions that may be connected with its fertility. This consequence raises the possibility that the fungus goes through a sexual cycle in its natural environment (Ikeda et al., 2002). Many researchers have reported the existence of RIP/RIP-like routes in *Aspergillus fumigatus, Fusarium graminearum, Leptosphaeria maculans,* and *M. grisea,* implying their prospective role in DNA methylation. This means that further investigation is crucial to elucidate its impending role in DNA methylation or DNA maintenance in plant fungal pathogens (Ikeda et al., 2002; Monroy and Sheppard, 2005; Fudal et al., 2009; Pomraning, 2012).

### Modifications and differences in histones

The highly conserved histone proteins can be changed through post-translational amendments. The nucleosome is made up of a cylinder of DNA coiled on top of an octameric complex of histones. Eukaryotic DNA is organized into higher-order chromatin threads through the repetitive assembly of nucleosome units. Epigenetic changes, which comprise the epigenetic code, are produced when specific chemical groups covalently link to particular dregs of histone proteins. The epigenetic code is made up of histone coding that can be decoded at the cellular stage through the protein’s contribution, both individually and collectively (Lennartsson and Ekwall, 2009). Further, it was noted that methylation and acetylation are the two main types of post-PTMs of histones in *M. oryzae*.

### Histone methylation and demethylation

**Figure 2:**
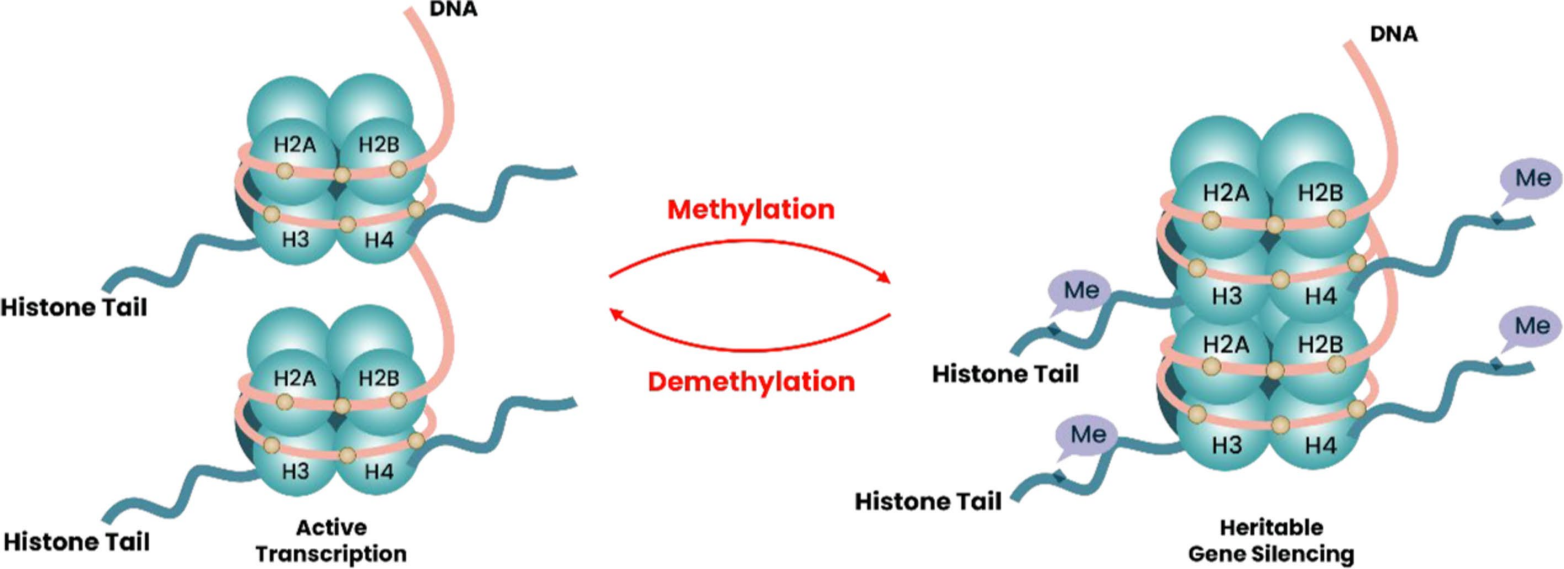
Diagram depicting the process of methylation and demethylation of histones. DNA twines on histone proteins, including H3/H4 and H2A/H2B dimers, create globular histone octamers, forming nucleosomes, the basic units of chromatin. Arginine and lysine residues in H3/4 tail undergo methylation, mediated by methyltransferases, and demethylation is modulated by demethylases. Histone methylation impacts chromatin structure, nucleosome structure, and gene expression. Gene expression is muted when the nucleosome structure gets packed due to the addition of methyl groups (Me) from arginine and residues of lysine in the tail, making transcription difficult. Demethylation of histones can open the structure by removing methyl groups (Me) from arginine and lysine residues, revealing binding spots for transcription factors and regulating gene activation.

Histone methylation and demethylation are crucial epigenetic modifications that regulate gene expression and maintain chromatin structure in eukaryotic cells. This process involves adding methyl groups to specific lysine or arginine residues on histone tails, which can either activate or repress gene transcription depending on the specific residue affected and the number of methyl groups attached (Separovich and Wilkins, 2021). Methylation occurs at specific lysine residues located within the N-terminal regions of histones H3-H4. In this situation, lysine methylation is commonly noted at N-terminal lysine residues K4, K9, K27, and K36, along with K79 within the H3 histone core and K20 within the H4 tail (Figure 2). Lysine methylations, specifically me1 (mono), me2 (di), and me3 (tri), collectively with their relative concentrations and sensitivity, are thought to influence gene transcription and repression (Sims et al., 2003). H3K4 trimethylation is typically linked to gene activation, while H3K9 trimethylation is indicative of a repressed chromatin state (Klose and Zhang, 2007). Aside from the lysine residue methylation activity, it is essential to note that arginine dregs can also undergo methylation at certain places, including H3-R2, H3-R8, H3-R17, H3-R26, and H4-R3 (Lorton and Shechter, 2019).

Histone methylation has been linked to both gene activation and repression in *M. oryzae*. For instance, in *M. oryzae*, a cellulase gene was activated in response to a substrate, with this activation being accelerated by histone methylation (Vu et al., 2013). The upregulation of the cellulase gene *MoCel7C* occurs upon contact with the host leaf surface, indicating the pathogen’s capacity for recognition upon encountering the host surface. The gene *MoSET1*, which is a counterpart to *SET1* in the species *Saccharomyces cerevisiae*, codes for enzymes known as histone lysine methyltransferases (KMTs). In *M. oryzae, MoSET1* is explicitly engaged in the methylation of histone H3 at the lysine 4 region (H3K4), specifically in the chromosomal area referred to as the *MoCel7C* gene. The *MoCel7C* gene experiences H3K4 methylation and subsequently, activation when exposed to an exogenous cellulosic substrate, especially a 2% concentration of carboxymethylcellulose (CMC). The knockout mutant of *MoSET1* in *M. oryzae* showed a dramatic reduction in the activity of the *MoCel7C* gene, correlating with its function of methylation in gene activation (Quoc and Bao Chau, 2017). A major change from the usual pattern was detected, wherein the lack of the substrate CMC led to an upregulation of *MoCel7C* expression in the *MoSET1* deletion mutant. This observation indicates that the process of H3K4 methylation is implied in the repression of genes under non-inducing circumstances.

The process of chromatin remodeling, which modifies the convenience of the promoter to the transcriptional mechanism in response to different environmental factors, can explain the dual effects (activation and suppression) of histone methylation. Similarly, another breakthrough effectively explained the involvement of *MoSET1* and various other KMTs in promoting the pathogenicity of *M. oryzae* (Minh et al., 2023). The interruption of *MoSET1*, a key element of the infection progression, led to a substantial reduction in condition and appressorium development within the rice blast pathogen. Consequently, this leads to the loss of pathogenicity towards the host plant. Pham et al. (2015) conducted an experiment that confirmed the impact of H3K4me methylation as an epigenetic marker in controlling the expression of infection-related genes in *M. oryzae*. Moreover, the aforementioned work presents empirical validation for the correlation between H3K4 methylation and the processes of gene activation and gene repression.

Pham et al. (2015) employed RNA-sequencing (RNA-seq) and chromatin immunoprecipitation-sequencing (ChIP-seq) techniques to unveil the discoveries that *MoSET1* in *M. oryzae* displayed direct impacts on gene activation and indirect impacts on gene repression. *MoSET1* was previously found to be involved in suppressing genes in non-inducing environments, therefore these findings are in line with those of Vu et al. (2013). Histone methylation activity can switch between activation and repression, providing additional confirmation of the link between environmental inputs and the periodicity of histone methylation. The pathogenicity of fungal infections might be better understood by looking at the environmental variables and situations that interact with HDM and HMT. The epigenetic process is complex, but a better understanding of the molecular route involved may help provide light on how to lessen the devastation caused by fungal infections in plants.

### Acetylation and deacetylation of histones

**Figure 3:**
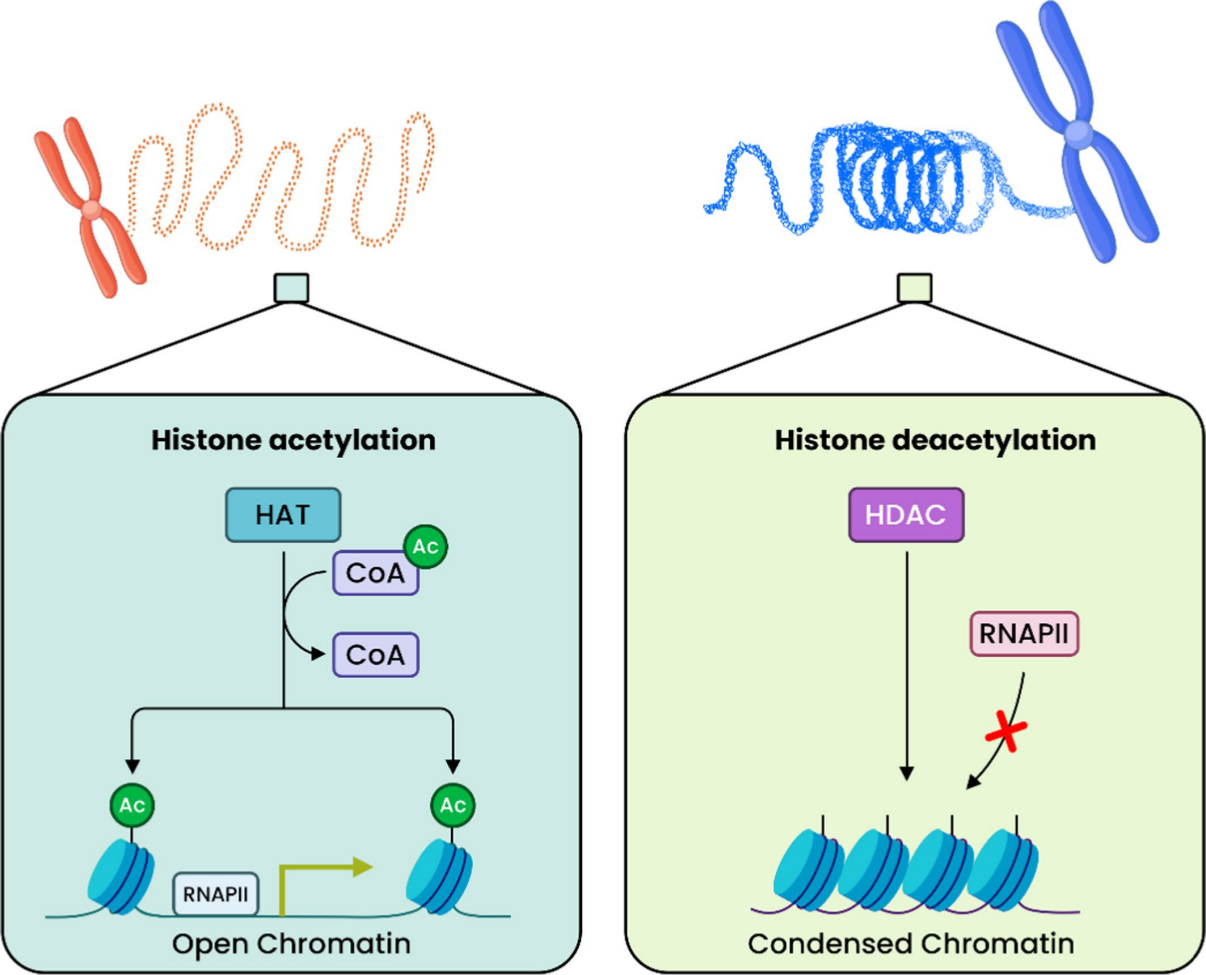
Histone acetylation and deacetylation are key processes in regulating gene expression. Acetylation involves attaching an acetyl group to the ε-position of lysine residues on histones, leading to a more open chromatin structure that facilitates gene transcription. Conversely, deacetylation removes the acetyl group from lysine residues, resulting in a more compact chromatin structure that is less accessible for active gene expression.

Histone acetylation and deacetylation are two of the most explored types of dynamic changes in biology (Tahir and Tian, 2021). HATs and the cofactor acetyl-CoA form protein complexes that acetylate numerous lysine residues, activating transcription. HATs can acetylate lysine residues, whereas HDACs counteract this process by deacetylating the amino group. The underlying DNA sequences become inaccessible by coiling and winding tightly around the histone after this process restores their positive charge (Figure 3) (Bannister and Kouzarides, 2011). Additionally, the utilization of web-based tools, such as ‘dbHiMo: an online genomics portal for histone-modifying enzymes’ (Choi et al., 2015), involves the use of hidden Markov models for the goal of classifying histone-modifying enzymes (HMEs) according to various features, including but not limited to domain structure.

The impact of HDACs on the virulence of the fungus *M. oryzae* has been examined, alongside HMTs and HDMs. In a study performed by Xu et al. (2022a,b), it was noted that the application of HDAC inhibitors derived from the Rpd3/Hda1 family of lysine deacetylases had a suppressive effect on appressorium production and pathogenicity in *M. oryzae*. The formerly cited finding highlights the significant contribution of HDACs to the differentiation of *M. oryzae* during asexual development. In *M. oryzae* the transducin beta-like gene *TIG1,* along with its counterparts in yeast and mammalian cells, has been detected as a component of the global histone deacetylase (HDAC) transcriptional corepressor complex. Ding et al. (2010) found that *TIG1* is necessary for pathogenicity and conidiogenesis of *M. oryzae*. The mutant was unable to produce invasive hyphae because it was hypersensitive to oxidative damage after *TIG1* was deleted. Besides, two histone deacetylases (HDACs), which have similarities to constituents of the yeast Set3 complex, were extracted from the filtered protein compounds linked with TIG1. This investigation underscores the importance of chromatin alterations in the context of plant infection and sheds light on the involvement of *TIG1*, a constituent of the HDAC complex, in the development of infection mechanisms used by *M. oryzae.* It has come to light that HDAC plays a role in phototropically enhancing asexual reproduction and pathogenicity in *M. oryzae* (Deng et al., 2015). *M. oryzae* has a protein named Twilight (TWL), which is under the control of its circadian clock and exhibits heightened activity during twilight hours. The transcription factor Tbf5, in probable cooperation with GCN5, functions downstream of the TWL-HDAC transcription relay to assist the translocation process. The creation of TWL is essential for virulence and asexual reproduction because it helps in the colonization of host systems. Taken as a whole, these inquiries stress the significance of further examination into this phenomenon from the perspective of pathogen physiology, alteration, and efficiency. This is demonstrated by the establishment of a clear relationship between histone acetylation and deacetylation and the virulent behavior of the fungal pathogen.

### Short RNA (sRNA)

Fungi utilize sRNAs to regulate gene expression and maintain genomic integrity. These small molecules, typically ranging from 20 to 30 nucleotides in length, are involved in various epigenetic processes that influence fungal development, virulence, and responses to environmental changes. Small interfering RNAs (siRNAs) play a crucial role in fungal RNA interference (RNAi) pathways by directing gene silencing. This repression can occur through mechanisms such as the degradation of target mRNAs or inhibition of translation. For instance, when organisms like *N. crassa* encounter transposable elements or repetitive DNA sequences, they produce siRNAs that help preserve genomic stability by preventing the expression of potentially harmful genes (Ouyang et al., 2021).

Post-transcriptional gene silencing (PTGS) refers to gene silencing mediated by sRNA and occurs in tandem with RNA interference (RNAi) components like sRNA-binding protein Argonaute, double-strand RNA endonuclease Dicer and RdRPs (RNA-dependent RNA polymerases) (Nicolás and Ruiz-Vázquez, 2013). Dicer uses the dsRNA as a template to create a small interfering RNA (siRNA) of around 20–30 bp in length. Subsequently, this siRNA is employed for loading onto the RNA-induced silencing complex (Svoboda, 2020), which in turn either disrupts or hinders the translation of the target messenger RNA (mRNA). Epigenetic alterations mediated by short RNA (sRNA) have been studied mostly in nonpathogenic fungi. However, this review will refrain from conducting an exhaustive analysis of epigenetic studies carried out in other fungal model systems. Instead, we provide a summary of established mechanisms of epigenetic regulation involving small RNAs in several fungal models. Subsequently, we explore their applicability to *M. oryzae*. In general, three RNA interference (RNAi)-based strategies for silencing genes in fungi are recognized. These mechanisms include the creation of heterochromatin, quelling, and meiotic silencing by unpaired DNA (MSUD).

### Heterochromatin formation

Heterochromatin development is crucial in fungal epigenetics, as it influences gene expression, genome stability, and defense mechanisms. It refers to tightly packed chromatin regions in fungi where genes remain transcriptionally silent (inactive). The formation of heterochromatin is regulated by several epigenetic processes, such as histone modifications, DNA methylation, and the recruitment of specific proteins that compact the chromatin structure (Jost et al., 2012). sRNA, in concert with RNAi-linked factors, binds to homologous DNA sequences, altering chromatin in a way that promotes gene silencing and heterochromatin formation. Studies have proved that sRNA and the elements of the RNAi machinery play a fundamental role in the establishment of heterochromatin in centromeres within the fungus *S. pombe* (Thon et al., 2005). Several studies have shown that H3K9me plays a role in gene silencing by recruiting Heterochromatin Protein1 (HP1) or its yeast ortholog SWI6 (Chicas et al., 2004; Shimada et al., 2009). The inability to elucidate the contribution of RNAi in heterochromatin formation was seen in *N. crassa* through the alteration of genetic variants of RdRP (Dang et al., 2011), and *S. cerevisiae* is missing the conventional RNAi components. These conclusions confirm an additional regulatory pathway based on RNA that affects epigenetic inheritance.

Kadotani et al. (2004) discovered the presence of two Dicer-like genes, namely *MoDcl1* and *MoDcl2*, in the rice blast fungus. These genes exhibit functional similarities to *dcl-1* and *dcl-2* genes found in the filamentous fungus *N. crassa.* In *M. oryzae*, *MoDcl2* functions in RNA silencing autonomously, separate from *MoDcl1*. Furthermore, it was found that elevating the expression of *MoDcl1* in the *MoDcl2* knockout mutant leads to the generation of siRNAs, suggesting the existence of an RNAi pathway that operates independently of *MoDcl2* (Kadotani et al., 2008). However, it is not yet clear what part these Dicer-like proteins play, if any, in the epigenetic control of *M. oryzae*. In addition, the sRNA profile of *M. oryzae* undergoes modifications in response to physiological stress and host conditions. This has been demonstrated through the analysis of sRNA transcriptome profiling of the pathogen under various physiological stresses, such as oxidative stress induced by paraquat, carbon starvation, minimal media conditions, and nitrogen starvation. The genes associated with the pathogenicity of *M. oryzae* were discovered to be a group whose transcription was increased in mutants of sRNA biosynthesis genes, including Dicer-like genes and RdRP genes (Raman et al., 2013). The silencing of TE-derived sequences by small interfering RNAs (sRNAs) in *Phytophthora infestans* led to the formation of heterochromatin at the affected locus, which, in turn, silenced sequences in both directions (Whisson et al., 2012). To date, there has been no conclusive evidence linking sRNA to the epigenetic regulation of growth or pathogenicity of *M. oryzae.* Nonetheless, these results underscore the significance of exploring sRNA-mediated epigenetic alterations in *M. oryzae* as a means to gain a more holistic comprehension of pathogen development, susceptibility to diseases, and interactions between the pathogen and its host.

### Quelling

In fungal epigenetics, quelling is one of the key processes involved in regulating gene expression. It is a post-transcriptional gene silencing mechanism, that was first discovered in the bread mold *N. crassa* (Fulci and Macino, 2007). This process leads to the silencing of both introduced and endogenous genes when extra copies of a gene are inserted. Similar to RNA interference (RNAi) in plants and animals, quelling relies on small RNA molecules to mediate gene silencing. Specifically, it utilizes small interfering RNAs (siRNAs) to degrade the messenger RNA (mRNA) of the targeted gene, thereby preventing its translation. The creation of heterochromatin and the control of genes are both known to be impacted by quelling (Tamaru and Selker, 2001). Qde-2-linked RNAs act to prevent protein translation when DNA damage occurs in *N. crassa.* The genes QDE-1-3 are responsible for encoding Argonaute-like protein, RdRP, and Rec-Qlike DNA helicase, respectively (Lee et al., 2009; Mann et al., 2023). QDE-1 plays a pivotal role in RNAi-mediated silencing by participating in the generation of aberrant RNA. To the best of our understanding, however, quelling has not been documented in *M. oryzae.* This implies the existence of alternative RNA-based silencing mechanisms that are likely more common in this pathosystem. Overall, quelling plays a crucial role in fungal biology, offering insights into gene regulation and genome protection in *N. crassa* and other fungal species. This mechanism may reveal important clues about how fungi control gene expression and safeguard their genomes.

### Meiotic silencing by unpaired DNA (MSUD)

Meiotic Silencing by Unpaired DNA (MSUD) is an intriguing epigenetic mechanism crucial for regulating gene expression in fungi during meiosis. This process, primarily observed in certain ascomycetes like *N. crassa,* plays a vital role in maintaining genomic integrity and ensuring proper sexual reproduction (Rhoades et al., 2021). MSUD is activated when homologous chromosomes fail to pair correctly during meiosis. The cellular machinery identifies the unpaired DNA sequences and initiates a silencing response. This mechanism involves the production of small RNAs that bind to specific regions of unpaired DNA, leading to the transcriptional silencing of the associated genes. This process relies on the participation of specific components, specifically SAD-1 to SAD-3, which facilitate the conversion of MSUD-induced aberrant RNA into double-stranded RNA (Shiu et al., 2001). Subsequently, Dcl1 processes the dsRNA into small RNAs, typically 20–25 nucleotides long. These small RNAs then interact with suppressors of meiotic silencing, such as SMS-2 (an Argonaute homolog), and QIP (an exonuclease), leading to the post-transcriptional suppression of similar genes (Aramayo and Selker, 2013).

Heterochromatin synthesis and normal centromeric and telomeric function need the aforementioned RNAi machinery (Billmyre et al., 2013). Except for QIP and dcl-1, which suggest a mutual ancestry that has evolved separately with time, the machinery necessary for quelling and MSUD functions through separate and distinct mechanisms. MSUD has been documented in several other fungal species other than *N. crassa,* including *F. oxysporum,* and the Basidiomycota (Billmyre et al., 2013; Chen et al., 2014). The prevalence of the sexual cycle in *M. oryzae* has not been documented in natural settings, suggesting that MSUD is uncommon in fungal plant pathogens. By delving deeper into MSUD, researchers can gain valuable insights into fungal biology and its impact on agriculture. This exploration could result in improved strategies for managing fungal plant ailments and enhancing crop resistance. Understanding the intricate relationship between genetics and epigenetics is necessary for advancing our knowledge of fungal behavior and developing innovative solutions in agriculture.

### Epigenetic mysteries of *M. oryzae* and associated fungal plant pathogens

Fungal epigenetics presents a wealth of untapped potential for scientific exploration, particularly in enhancing agricultural practices and elucidating pathogenic mechanisms. By investigating the epigenetic regulation of gene expression, researchers can uncover the intricate processes that drive fungal virulence, host-pathogen interactions, and stress responses. This knowledge not only facilitates the development of more resilient agricultural methods but also informs innovative strategies for managing fungal diseases in crops (Kramer et al., 2023). Additionally, examining the role of epigenetics in regulating transposable elements and ensuring genomic stability can deepen our understanding of fungal evolution and adaptation. Ultimately, harnessing the complexities of fungal epigenetics could lead to advancements in agricultural productivity and food security, paving the way for novel therapeutic approaches and sustainable solutions for crop protection.

The numerous unanswered questions surrounding the epigenetics of fungal plant diseases provide valuable insights into fungal biology and its implications for agriculture. Comprehensive investigations have been conducted on fungal model organisms, namely *N. crassa* and *Aspergillus nidulans*, in terms of their epigenetic characteristics (Lafontaine, 2010; Zhao et al., 2022). Nevertheless, our understanding of how epigenetics influences the regulation of virulence and the developmental processes involved in fungal infections remains limited. These epigenetic changes studied in *M. oryzae* and related pathogens, have been less investigated but still have a substantial impact on the aggressiveness of fungal plant pathogens.

Stergiopoulos and de Wit (2009) investigated the role of specific avirulence genes in fungi which are responsible for encoding effector protein, a known virulence factor in fungi. Pathogen-effector proteins are imperative for inducing infection in host plants, yet the host’s immune system is capable of making them avirulent. Avirulent genes (Avr) are smart enough to evade host immune responses by undergoing mutations or multiple translocations as reported by Chuma et al. (2011) and Raffaele and Kamoun (2012). Furthermore, from a pathogen’s perspective, the epigenetic switches employ the effectors to increase virulence. The model provided by Gijzen et al. (2014) elucidates the possible impact of alterations in epigenetic equilibrium on transposon activity and Avr gene expression, eventually leading to alterations in virulence. Soyer et al. (2014) researched *L. maculans*, a fungal organism that belongs to the phylum Ascomycota, and demonstrated that H3K9me3 plays a distinctive role in the epigenetic regulation of effector gene expression. Epigenetic mutations were triggered in the pathogen during its transition from a culture without other organisms to its primary infection site. These modifications caused shifts in the expression of effector genes inside the adenine-thymine (AT) isochore sites. The process of RNA interference (RNAi) was employed to silence the expression of DIM-5 and HP1, two decisive factors implicated in the assembly and maintenance of heterochromatin in *L. maculans*. This silencing followed in the upregulation of genes encoding small secreted proteins (SSPs) within AT-rich isochores in the transformed organisms when grown in a deliberate culture environment. Alterations in the environment have the potential to activate an epigenetic process that can either activate or suppress the occurrence of fungal pathogens through the course of chromatin remodeling.

## Etiology, host-pathogen interactions, and epidemiology based on epigenetics

Adaptive evolution, both in prokaryotes and eukaryotes, is hypothesized to be strongly influenced by epigenetics. In general, DNMTs recognize particular patterns of DNA sequence for methylation via their target recognition domain (TRD). The methylation pattern of the entire genome is reported to be altered in bacteria due to the movement of TRDs across non-orthogonal genes and sometimes within genes (Furuta and Kobayashi, 2012), which ultimately results in global changes in gene expression. Therefore, it is hypothesized that adaptive evolution in bacterial pathogens aims at the diverse methylome rather than the diverse genome. Fungal genomes, on the other hand, are not heavily methylated like those of plants and animals. He et al. (2020) reveal that *M. oryzae* has DNA methylation at some cytosine locations, albeit at relatively low levels. This leaves the ultimate significance of methylome in driving adaptive evolution and the impact of evolutionary selection forces unclear. In contrast, our proposition posits that the analysis of histone methylation patterns in fungal isolates from diverse geographic regions could contribute to the reconstruction of the evolutionary trajectory and spatial dispersion of pathogenic fungi.

Fungal pathogens can evade the host immune system, in part, by modifying their effector proteins. The evasion of host defenses is influenced by various factors, including sequence alterations or loss of virulence genes, as well as the involvement of a transgenerational epigenetic mechanism. Epigenetic variation, specifically through the participation of transposable elements can impact the control of virulence in fungal pathogens. TEs are recognized for their significant role in shaping phenotypic diversity and the co-evolution of interactions between hosts and pathogens (Kasuga and Gijzen, 2013). Future studies of infectious diseases would benefit greatly from epigenetic epidemiology due to the high epigenetic implication in host-pathogen interactions (Gómez-Díaz et al., 2012). While there have been advancements in comprehending the molecular underpinnings of epigenetics and variation, there has been limited exploration into the potential role of epigenetics in interactions between hosts and pathogens.

### Epigenetic regulation and chromatin remodeling gene mutant strains

The effect of genes involved in chromatin structure on fungal growth, physiology, metabolism, and the generation of chemicals has been the focus of several studies. Using dominant selection markers to replace or inactivate genes has yielded useful data. We describe some of the most impressive studies below.

### Calcarisporium arbuscula

The mushroom-endophytic fungus *Calcarisporium arbuscula* has been identified as a possible source of PKEs. Changes in growth, hyphae shape, and sporulation were seen after the deletion of *hdaA*, which encodes a histone deacetylase (HDAC) in *C. arbuscula.* Several novel natural compounds with distinct chemical structures and biosynthetic routes were identified using a liquid chromatography/mass spectrometry study on a crude extract from the mutant strain (Mao et al., 2015).

### *Pestalotiopsis microspora* and *P. fici*

The endophytic fungus *Pestalotiopsis microspora* has the potential to be used in the biological degradation of plastics since it can break down polyester and polyurethane (Liu et al., 2023). The impairment of conidiation, melanin pigmentation, and secondary metabolites (SMs) synthesis was seen as a result of deleting the hat1 gene encoding a fungal histone acetyltransferase (Zhang et al., 2016). Another endophyte whose SMs production is epigenetically controlled is *Pestalotiopsis fici*. Wu et al. (2016) conducted gene disruptions involving *PfCcla,* a gene associated with histone methylation, and *PfH-daA,* a gene associated with histone deacetylation. In comparison to these wild-type strains, the HPLC profiles of raw extracts from the mutants showed the presence of 15 more polyketides.

### Fusarium graminearum

Connolly et al. (2013) removed the potential histone methyltransferase KMT6 gene from the wheat pathogen *F. graminearum*. Western blotting using a variety of antibodies confirmed that H3K27me3 tri-methylation on histone 3 was absent. Long-term investigations on Ryan race test tubes found that mutant strains had a twofold slower progression, an intense orange coloring, and proved to be sterile in crossing trials (Connolly et al., 2013). Fifteen percent to 30 % of the genes in *F. graminearum* were repressed because of the deletion of the *kmt6* gene. The majority of these genes were found to be associated with SM biosynthesis clusters, and they included those needed for the synthesis of pigments, including aurofusarin, neurosporaxanthin, and torulene, as well as the mycotoxin fusarin C. Nitrogen levels serve as a well-established regulator of secondary metabolite (SM) synthesis in *Fusarium* species. Interestingly, when the H3K27me3 mark was removed, it revealed additional cryptic gene clusters. Despite the *kmt6* mutant strain demonstrating hypovirulence in a wounded tomato infection experiment, it displayed upregulation of genes related to potentially virulent components secreted by the fungus and genes involved in breaking down plant cell walls. A reconstituted strain exhibited nearly all the characteristics of wild-type organisms. Deletion of the *Set1* gene in *F. graminearum* led to deficiencies in virulence on wheat flower heads and in the production of aurofusarin and deoxynivalenol. *Set1* encodes a histone methyltransferase capable of promoting mono-, di-, and trimethylation of H3K4. However, in 2015, it was found that the organisms exhibited an increased resistance to chemicals capable of damaging cell walls (Liu et al., 2015).

### Ustilago maydis

*Ustilago maydis*, the basidiomycete responsible for maize smut, has been investigated intensively as a model organism for interactions between plants and pathogens throughout the 20th century (Yu et al., 2023). In a study conducted by Martínez-Soto et al. (2015), transcriptome studies were carried out using the *U. maydis* strain that had a deficiency in a gene responsible for producing histone acetyltransferase (*GCN5*). This choice was based on their earlier findings, which showed that mutant strains lacking the Δ*Umgcn5* gene were unable to cause disease and did not produce teliospores (González-Prieto et al., 2014). The overall transcription profile of wild-type vs. Δ*Umgcn5* strains was revealed to be drastically different, with 1,203 genes showing differential expression between the two strains. Most of these genes play roles in mycelial expansion and pathogenicity.

### CRISPR-based epigenetic modifications

A novel strategy for treating plant diseases can be achieved through CRISPR-based epigenetic modifications in phytopathogens. These modifications specifically target the gene expression of pathogens without altering their DNA. Phytopathogens, which include fungi, bacteria, and viruses, are accountable for significant crop losses worldwide. CRISPR technology, utilizing a catalytically inactive form of Cas9 (dCas9), enables precise regulation of key pathogenicity genes in these organisms. By combining dCas9 with epigenetic modifiers such as DNA methyltransferases or histone deacetylases, it is possible to downregulate genes involved in pathogen virulence or survival. This approach effectively reduces the pathogen’s capability to infect and damage crops without causing permanent genetic changes (Muñoz et al., 2019).

CRISPR-based epigenetic changes can silence genes related to toxin production, spore formation, or infection mechanisms in bacterial and fungal plant pathogens (Li et al., 2021). For instance, methylating the promoters of genes essential for releasing virulence proteins can decrease the pathogen’s capacity to cause disease. This targeted, non-genomic approach to managing pathogen behavior offers a significant advantage over traditional methods like fungicides and antibiotics by focusing on specific gene pathways and lowering the risk of resistance development. CRISPR-based techniques also facilitate a deeper understanding of gene function in plant pathogens, offering flexibility due to the reversibility of epigenetic changes. By dynamically switching specific genes on or off, scientists can analyze the role of different virulence factors in real-time, which could lead to discovering new directions to control diseases. In agriculture, this approach could be used to engineer crops with improved resistance to multiple diseases, providing a more sustainable and eco-friendly substitute to chemical treatments, and facing fewer regulatory hurdles compared to genetically modified organisms (Kumar, 2019).

## Conclusion

The ability of plant pathogens to persist in a wide variety of host environments has often been linked to epigenetics. While significant advancements have been made in unraveling the molecular mechanisms underlying epigenetics and the diversity of infections, the field of comparative epigenetics in pathogenic organisms remains largely underexplored. Given the critical role of epigenetic mechanisms in the success, persistence, and interactions between pathogens and their hosts, there is substantial potential for advancing our understanding of infectious diseases through the development of epigenetic epidemiology. Understanding the epigenetic alterations that pathogens undergo during infection, as well as their intracellular and intercellular growth, could significantly enhance our strategies for combating plant diseases.

### Future directions

Future research should focus on comparing the epigenetic variations observed in different growth stages, morphologies, and environments among various categories of plant pathogens to identify specific patterns of epigenetic modifications associated with plant diseases. Although some epigenetic modifications in *M. oryza* and their implications for other fungal plant diseases have been explored, many uncharted areas within *M. oryzae* warrant further investigation. The limited number of scholarly articles dedicated to fungal plant pathogens hampers our ability to synthesize and fully understand these phenomena. Therefore, more studies on the removal or preservation of epigenetic modifications are essential to gain deeper insights into the life cycle of fungi and their evolutionary adaptability. Additionally, identifying common epigenetic changes among diverse pathogens from different geographic regions can enhance our understanding of effective pathogenicity and reveal epigenetic trends during pathogenesis.

## References

[ref1] Aghcheh R. K. Kubicek C. P. (2015). Epigenetics as an emerging tool for improvement of fungal strains used in biotechnology. Appl. Microbiol. Biotechnol. 99, 6167–6181. doi: 10.1007/s00253-015-6763-2, PMID: 26115753

[ref2] Ali S. Laurie J. D. Linning R. Cervantes-Chávez J. A. Gaudet D. Bakkeren G. (2014). An immunity-triggering effector from the barley smut fungus *Ustilago hordei* resides in an Ustilaginaceae-specific cluster bearing signs of transposable element-assisted evolution. PLoS Pathog. 10:e1004223. doi: 10.1371/journal.ppat.1004223, PMID: 24992661 PMC4081816

[ref4] Anwaar H. A. Perveen R. Mansha M. Z. Abid M. Sarwar Z. M. Aatif H. M.. (2020). Assessment of grain yield indices in response to drought stress in wheat (*Triticum aestivum* L.). Saudi J. Biol. sciences 27, 1818–1823. doi: 10.1016/j.sjbs.2019.12.009, PMID: 32565701 PMC7296489

[ref5] Aramayo R. Selker E. U. (2013). *Neurospora crassa,* a model system for epigenetics research. Cold Spring Harb. Perspect. Biol. 5:a017921. doi: 10.1101/cshperspect.a017921, PMID: 24086046 PMC3783048

[ref6] Aslam H. M. U. Gleason M. L. Abbas A. Ul Abdin Z. Amrao L. (2019). Molecular characterization of *Magnaporthe oryzae* in Punjab, Pakistan and its *in vitro* suppression by fungicides and botanicals. Int. J. Agric. Biol. 22, 1459–1466. doi: 10.17957/IJAB/15.1222

[ref7] Aslam H. Khan N. Hussain S. Ali Y. Raheel M. Shahzad R.. (2022). First report of Brown leaf spot of Rice (*Oryza sativa*) caused by *Bipolaris sorokiniana* in Pakistan. Plant Dis. 106:1750. doi: 10.1094/PDIS-05-21-1097-PDN, PMID: 34798785

[ref8] Bannister A. J. Kouzarides T. (2011). Regulation of chromatin by histone modifications. Cell Res. 21, 381–395. doi: 10.1038/cr.2011.22, PMID: 21321607 PMC3193420

[ref9] Billmyre R. B. Calo S. Feretzaki M. Wang X. Heitman J. (2013). RNAi function, diversity, and loss in the fungal kingdom. Chromosom. Res. 21, 561–572. doi: 10.1007/s10577-013-9388-2 PMC3874831 24173579

[ref10] Castonguay E. Angers B. (2012). The key role of epigenetics in the persistence of asexual lineages. Genet. Res. Int. 2012, 1–9. doi: 10.1155/2012/534289 PMC3335536 22567390

[ref11] Chadha S. (2021). Analysis of genetic variations and genomic instabilities in *Magnaporthe oryzae*. Methods Mol. Biol. 2356, 211–224. doi: 10.1007/978-1-0716-1613-0_17 34236689

[ref12] Chang Z. Yadav V. Lee S. C. Heitman J. (2019). Epigenetic mechanisms of drug resistance in fungi. Fungal Genet. Biol. 132:103253. doi: 10.1016/j.fgb.2019.103253, PMID: 31325489 PMC6858951

[ref13] Chen R. Jiang N. Jiang Q. Sun X. Wang Y. Zhang H.. (2014). Exploring microRNA-like small RNAs in the filamentous fungus *Fusarium oxysporum*. PLoS One 9:e104956. doi: 10.1371/journal.pone.0104956, PMID: 25141304 PMC4139310

[ref14] Chen Z. Li S. Subramaniam S. Shyy J. Y.-J. Chien S. (2017). Epigenetic regulation: a new frontier for biomedical engineers. Annu. Rev. Biomed. Eng. 19, 195–219. doi: 10.1146/annurev-bioeng-071516-044720, PMID: 28301736

[ref15] Chicas A. Cogoni C. Macino G. (2004). RNAi-dependent and RNAi-independent mechanisms contribute to the silencing of RIPed sequences in *Neurospora crassa*. Nucleic Acids Res. 32, 4237–4243. doi: 10.1093/nar/gkh764, PMID: 15302921 PMC514385

[ref16] Choi J. Kim K.-T. Huh A. Kwon S. Hong C. Asiegbu F. O.. (2015). dbHiMo: a web-based epigenomics platform for histone-modifying enzymes. Database 2015:bav052. doi: 10.1093/database/bav052 26055100 PMC4460409

[ref17] Chuma I. Isobe C. Hotta Y. Ibaragi K. Futamata N. Kusaba M.. (2011). Multiple translocation of the AVR-Pita effector gene among chromosomes of the rice blast fungus *Magnaporthe oryzae* and related species. PLoS Pathog. 7:e1002147. doi: 10.1371/journal.ppat.1002147, PMID: 21829350 PMC3145791

[ref18] Clapier C. R. Cairns B. R. (2009). The biology of chromatin remodeling complexes. Annu. Rev. Biochem. 78, 273–304. doi: 10.1146/annurev.biochem.77.062706.153223 19355820

[ref19] Connolly L. R. Smith K. M. Freitag M. (2013). The *Fusarium graminearum* histone H3 K27 methyltransferase KMT6 regulates development and expression of secondary metabolite gene clusters. PLoS Genet. 9:e1003916. doi: 10.1371/journal.pgen.1003916, PMID: 24204317 PMC3814326

[ref20] Cutter Dipiazza A. R. Taneja N. Dhakshnamoorthy J. Wheeler D. Holla S. Grewal S. I. (2021). Spreading and epigenetic inheritance of heterochromatin require a critical density of histone H3 lysine 9 tri-methylation. Proc. Natl. Acad. Sci. 118:e2100699118. doi: 10.1073/pnas.2100699118, PMID: 34035174 PMC8179192

[ref21] Dang Y. Yang Q. Xue Z. Liu Y. (2011). RNA interference in fungi: pathways, functions, and applications. Eukaryot. Cell 10, 1148–1155. doi: 10.1128/EC.05109-11, PMID: 21724934 PMC3187057

[ref22] Deng Y. Z. Qu Z. Naqvi N. I. (2015). Twilight, a novel circadian-regulated gene, integrates phototropism with nutrient and redox homeostasis during fungal development. PLoS Pathog. 11:e1004972. doi: 10.1371/journal.ppat.1004972, PMID: 26102503 PMC4478003

[ref23] Ding S.-L. Liu W. Iliuk A. Ribot C. Vallet J. Tao A.. (2010). The tig1 histone deacetylase complex regulates infectious growth in the rice blast fungus *Magnaporthe oryzae*. Plant Cell 22, 2495–2508. doi: 10.1105/tpc.110.074302, PMID: 20675574 PMC2929099

[ref24] Erkina T. Y. Zou Y. Freeling S. Vorobyev V. Erkine A. M. (2010). Functional interplay between chromatin remodeling complexes RSC, SWI/SNF and ISWI in regulation of yeast heat shock genes. Nucleic Acids Res. 38, 1441–1449. doi: 10.1093/nar/gkp1130, PMID: 20015969 PMC2836563

[ref26] Freitag M. Hickey P. C. Khlafallah T. K. Read N. D. Selker E. U. (2004). HP1 is essential for DNA methylation in *Neurospora*. Mol. Cell 13, 427–434. doi: 10.1016/S1097-2765(04)00024-3, PMID: 14967149

[ref27] Fudal I. Ross S. Brun H. Besnard A.-L. Ermel M. Kuhn M.-L.. (2009). Repeat-induced point mutation (RIP) as an alternative mechanism of evolution toward virulence in *Leptosphaeria maculans*. Mol. Plant-Microbe Interact. 22, 932–941. doi: 10.1094/MPMI-22-8-0932, PMID: 19589069

[ref28] Fulci V. Macino G. (2007). Quelling: post-transcriptional gene silencing guided by small RNAs in Neurospora crassa. Curr. Opin. Microbiol. 10, 199–203. doi: 10.1016/j.mib.2007.03.016, PMID: 17395524

[ref29] Furuta Y. Kobayashi I. (2012). Mobility of DNA sequence recognition domains in DNA methyltransferases suggests epigenetics-driven adaptive evolution. Mob. Genet. Elem. 2, 292–296. doi: 10.4161/mge.23371, PMID: 23481556 PMC3575425

[ref30] Gijzen M. Ishmael C. Shrestha S. D. (2014). Epigenetic control of effectors in plant pathogens. Front. Plant Sci. 5:638. doi: 10.3389/fpls.2014.00638 25429296 PMC4228847

[ref31] Gómez-Díaz E. Jorda M. Peinado M. A. Rivero A. (2012). Epigenetics of host–pathogen interactions: the road ahead and the road behind. PLoS Pathog. 8:e1003007. doi: 10.1371/journal.ppat.1003007, PMID: 23209403 PMC3510240

[ref32] González-Prieto J. M. Rosas-Quijano R. Domínguez A. Ruiz-Herrera J. (2014). The UmGcn5 gene encoding histone acetyltransferase from *Ustilago maydis* is involved in dimorphism and virulence. Fungal Genet. Biol. 71, 86–95. doi: 10.1016/j.fgb.2014.09.002, PMID: 25242418

[ref33] Grewal S. I. Elgin S. C. (2007). Transcription and RNA interference in the formation of heterochromatin. Nature 447, 399–406. doi: 10.1038/nature05914, PMID: 17522672 PMC2950806

[ref34] Hammond T. M. Xiao H. Boone E. C. Decker L. M. Lee S. A. Perdue T. D.. (2013). Novel proteins required for meiotic silencing by unpaired DNA and siRNA generation in *Neurospora crassa*. Genetics 194, 91–100. doi: 10.1534/genetics.112.148999, PMID: 23502675 PMC3632485

[ref35] He C. Zhang Z. Li B. Tian S. (2020). The pattern and function of DNA methylation in fungal plant pathogens. Microorganisms 8:227. doi: 10.3390/microorganisms8020227, PMID: 32046339 PMC7074731

[ref36] Ikeda K. I. Nakayashiki H. Kataoka T. Tamba H. Hashimoto Y. Tosa Y.. (2002). Repeat-induced point mutation (RIP) in *Magnaporthe grisea*: implications for its sexual cycle in the natural field context. Mol. Microbiol. 45, 1355–1364. doi: 10.1046/j.1365-2958.2002.03101.x 12207702

[ref37] Ikeda K. Nakayashiki H. Takagi M. Tosa Y. Mayama S. (2001). Heat shock, copper sulfate and oxidative stress activate the retrotransposon MAGGY resident in the plant pathogenic fungus *Magnaporthe grisea*. Mol. Gen. Genomics. 266, 318–325. doi: 10.1007/s004380100560, PMID: 11683275

[ref38] Ikeda K.-I. Van Vu B. Kadotani N. Tanaka M. Murata T. Shiina K.. (2013). Is the fungus *Magnaporthe* losing DNA methylation? Genetics 195, 845–855. doi: 10.1534/genetics.113.155978 23979580 PMC3813868

[ref39] Itoh Y. (2020). Drug discovery researches on modulators of lysine-modifying enzymes based on strategic chemistry approaches. Chem. Pharm. Bull. 68, 34–45. doi: 10.1248/cpb.c19-00741, PMID: 31902900

[ref40] Jones P. A. Liang G. (2009). Rethinking how DNA methylation patterns are maintained. Nat. Rev. Genet. 10, 805–811. doi: 10.1038/nrg2651, PMID: 19789556 PMC2848124

[ref41] Jost K. L. Bertulat B. Cardoso M. C. (2012). Heterochromatin and gene positioning: inside, outside, any side? Chromosoma 121, 555–563. doi: 10.1007/s00412-012-0389-2, PMID: 23090282 PMC3501169

[ref42] Kadotani N. Murata T. Quoc N. B. Adachi Y. Nakayashiki H. (2008). Transcriptional control and protein specialization have roles in the functional diversification of two dicer-like proteins in *Magnaporthe oryzae*. Genetics 180, 1245–1249. doi: 10.1534/genetics.108.093922, PMID: 18791228 PMC2567371

[ref43] Kadotani N. Nakayashiki H. Tosa Y. Mayama S. (2004). One of the two dicer-like proteins in the filamentous fungi *Magnaporthe oryzae* genome is responsible for hairpin RNA-triggered RNA silencing and related small interfering RNA accumulation. J. Biol. Chem. 279, 44467–44474. doi: 10.1074/jbc.M408259200, PMID: 15304480

[ref44] Kasuga T. Gijzen M. (2013). Epigenetics and the evolution of virulence. Trends Microbiol. 21, 575–582. doi: 10.1016/j.tim.2013.09.003 24095304

[ref45] Klose R. J. Zhang Y. (2007). Regulation of histone methylation by demethylimination and demethylation. Nat. Rev. Mol. Cell Biol. 8, 307–318. doi: 10.1038/nrm2143, PMID: 17342184

[ref46] Kramer H. M. Cook D. E. Seidl M. F. Thomma B. P. (2023). Epigenetic regulation of nuclear processes in fungal plant pathogens. PLoS Pathog. 19:e1011525. doi: 10.1371/journal.ppat.1011525, PMID: 37535497 PMC10399791

[ref47] Kronholm I. Johannesson H. Ketola T. (2016). Epigenetic control of phenotypic plasticity in the filamentous fungus *Neurospora crassa*. G3 6, 4009–4022. doi: 10.1534/g3.116.033860 27694114 PMC5144970

[ref48] Kumar S. (2019). Epigenetics and epigenomics for crop improvement: current opinion. Adv. Biotechnol. Microbiol. 14:555879. doi: 10.19080/AIBM.2019.14.555879

[ref49] Kumar S. Chinnusamy V. Mohapatra T. (2018). Epigenetics of modified DNA bases: 5-methylcytosine and beyond. Front. Genet. 9:640. doi: 10.3389/fgene.2018.00640, PMID: 30619465 PMC6305559

[ref50] Lafontaine D. (2010). Genetic and epigenetic control of a nonself recognition complex in N. crassa. Ottawa, Ontario: Carleton University. UMI Dissertation Publishing. Library and Archives Canada. Published Heritage Branch.

[ref51] Lee H.-C. Chang S.-S. Choudhary S. Aalto A. P. Maiti M. Bamford D. H.. (2009). qiRNA is a new type of small interfering RNA induced by DNA damage. Nature 459, 274–277. doi: 10.1038/nature08041, PMID: 19444217 PMC2859615

[ref52] Lennartsson A. Ekwall K. (2009). Histone modification patterns and epigenetic codes. Biochim. Biophys. 1790, 863–868. doi: 10.1016/j.bbagen.2008.12.006 19168116

[ref53] Li X. Huang L. Pan L. Wang B. Pan L. (2021). CRISPR/dCas9-mediated epigenetic modification reveals differential regulation of histone acetylation on *aspergillus Niger* secondary metabolite. Microbiol. Res. 245:126694. doi: 10.1016/j.micres.2020.126694, PMID: 33482403

[ref54] Li W. Wang Y. Zhu J. Wang Z. Tang G. Huang B. (2017). Differential DNA methylation may contribute to temporal and spatial regulation of gene expression and the development of mycelia and conidia in entomopathogenic fungus *Metarhizium robertsii*. Fungal Biol. 121, 293–303. doi: 10.1016/j.funbio.2017.01.002, PMID: 28215355

[ref55] Liu Y. Liu N. Yin Y. Chen Y. Jiang J. Ma Z. (2015). Histone H3K4 methylation regulates hyphal growth, secondary metabolism and multiple stress responses in *Fusarium graminearum*. Environ. Microbiol. 17, 4615–4630. doi: 10.1111/1462-2920.12993, PMID: 26234386

[ref56] Liu J. Zeng Q. Lei H. Xin K. Xu A. Wei R.. (2023). Biodegradation of polyester polyurethane by *Cladosporium* sp. P7: evaluating its degradation capacity and metabolic pathways. J. Hazard. Mater. 448:130776. doi: 10.1016/j.jhazmat.2023.130776, PMID: 36706489

[ref57] Lorton B. M. Shechter D. (2019). Cellular consequences of arginine methylation. Cell. Mol. Life Sci. 76, 2933–2956. doi: 10.1007/s00018-019-03140-2, PMID: 31101937 PMC6642692

[ref58] Madhani H. D. (2021). Unbelievable but true: epigenetics and chromatin in fungi. Trends Genet. 37, 12–20. doi: 10.1016/j.tig.2020.09.016, PMID: 33092902 PMC8994648

[ref59] Malagnac F. Silar P. (2011). “Epigenetics of eukaryotic microbes” in Handbook of epigenetics. ed. Tollefsbol T. O. (Amsterdam: Elsevier), 185–201.

[ref60] Mann C. W. Sawyer A. Gardiner D. M. Mitter N. Carroll B. J. Eamens A. L. (2023). RNA-based control of fungal pathogens in plants. Int. J. Mol. Sci. 24:12391. doi: 10.3390/ijms241512391, PMID: 37569766 PMC10418863

[ref61] Mao X. M. Xu W. Li D. Yin W. B. Chooi Y. H. Li Y. Q.. (2015). Epigenetic genome mining of an endophytic fungus leads to the pleiotropic biosynthesis of natural products. Angew. Chem. 127, 7702–7706. doi: 10.1002/ange.201502452 PMC4487767 26013262

[ref62] Martínez-Soto D. Gonzalez-Prieto J. M. Ruiz-Herrera J. (2015). Transcriptomic analysis of the GCN5 gene reveals mechanisms of the epigenetic regulation of virulence and morphogenesis in *Ustilago maydis*. FEMS Yeast Res. 15:fov055. doi: 10.1093/femsyr/fov055, PMID: 26126523

[ref63] Martin-Urdiroz M. Oses-Ruiz M. Ryder L. S. Talbot N. J. (2016). Investigating the biology of plant infection by the rice blast fungus *Magnaporthe oryzae*. Fungal Genet. Biol. 90, 61–68. doi: 10.1016/j.fgb.2015.12.009 26703899

[ref1000] Martin F. (2017). Molecular mycorrhizal symbiosis. Wiley Online Library. John Wiley & Sons, Inc.

[ref64] Meyer V. Andersen M. R. Brakhage A. A. Braus G. H. Caddick M. X. Cairns T. C.. (2016). Current challenges of research on filamentous fungi in relation to human welfare and a sustainable bio-economy: a white paper. Fungal Biol. Biotechnol. 3, 1–17. doi: 10.1186/s40694-016-0024-8 28955465 PMC5611618

[ref65] Mierziak J. Wojtasik W. (2024). Epigenetic weapons of plants against fungal pathogens. BMC Plant Biol. 24:175. doi: 10.1186/s12870-024-04829-8, PMID: 38443788 PMC10916060

[ref66] Minh D. N. Tsukahara Y. Thach D. A. Ikeda K.-I. Nakayashiki H. (2023). MoSET1-dependent transcription factors regulate different stages of infection-related morphogenesis in Pyricularia oryzae. J. Gen. Plant Pathol. 89, 77–83. doi: 10.1007/s10327-022-01111-3

[ref67] Monroy F. Sheppard D. C. (2005). Taf1: a class II transposon of *aspergillus fumigatus*. Fungal Genet. Biol. 42, 638–645. doi: 10.1016/j.fgb.2005.04.003, PMID: 15896988

[ref68] Morlando M. Ballarino M. Fatica A. Bozzoni I. (2014). The role of long noncoding RNAs in the epigenetic control of gene expression. ChemMedChem 9, 505–510. doi: 10.1002/cmdc.201300569 24488863

[ref1001] Muñoz I. V. Sarrocco S. Malfatti L. Baroncelli R. Vannacci G. (2019). CRISPR-Cas for fungal genome editing: a new tool for the management of plant diseases. Front. Plant Sci. 10:135. 30828340 10.3389/fpls.2019.00135 PMC6384228

[ref69] Na R. Yu D. Chapman B. P. Zhang Y. Kuflu K. Austin R.. (2014). Genome re-sequencing and functional analysis places the *Phytophthora sojae* avirulence genes Avr1c and Avr1a in a tandem repeat at a single locus. PLoS One 9:e89738. doi: 10.1371/journal.pone.0089738, PMID: 24586999 PMC3933651

[ref70] Nakayashiki H. Ikeda K. Hashimoto Y. Tosa Y. Mayama S. (2001). Methylation is not the main force repressing the retrotransposon MAGGY in *Magnaporthe grisea*. Nucleic Acids Res. 29, 1278–1284. doi: 10.1093/nar/29.6.1278, PMID: 11238993 PMC29754

[ref71] Nicolás F. E. Ruiz-Vázquez R. M. (2013). Functional diversity of RNAi-associated sRNAs in fungi. Int. J. Mol. Sci. 14, 15348–15360. doi: 10.3390/ijms140815348, PMID: 23887655 PMC3759863

[ref72] Ouyang S.-Q. Park G. Ji H.-M. Borkovich K. A. (2021). Small RNA isolation and library construction for expression profiling of small RNAs from Neurospora crassa and *Fusarium oxysporum* and analysis of small RNAs in *Fusarium oxysporum*-infected plant root tissue. Methods Protoc. 2170, 199–212. doi: 10.1007/978-1-0716-0743-5_14 32797460

[ref73] Pham K. T. M. Inoue Y. Vu B. V. Nguyen H. H. Nakayashiki T. Ikeda K.-I.. (2015). MoSET1 (histone H3K4 methyltransferase in *Magnaporthe oryzae*) regulates global gene expression during infection-related morphogenesis. PLoS Genet. 11:e1005385. doi: 10.1371/journal.pgen.1005385 26230995 PMC4521839

[ref74] Poças-Fonseca M. J. Cabral C. G. Manfrão-Netto J. H. C. (2020). Epigenetic manipulation of filamentous fungi for biotechnological applications: a systematic review. Biotechnol. Lett. 42, 885–904. doi: 10.1007/s10529-020-02871-8, PMID: 32246346

[ref75] Pomraning K. R. (2012). Characterization of *Neurospora crassa* and *Fusarium graminearum* mutants defective in repeat-induced point mutation. Oregon State University. UMI Dissertation Publishing. Ann Arbor, United States: ProQuest LLC.

[ref76] Popay T. M. Dixon J. R. (2022). Coming full circle: on the origin and evolution of the looping model for enhancer–promoter communication. J. Biol. Chem. 298:102117. doi: 10.1016/j.jbc.2022.102117, PMID: 35691341 PMC9283939

[ref77] Quoc N. B. Bao Chau N. N. (2017). The role of cell wall degrading enzymes in pathogenesis of *Magnaporthe oryzae*. Curr. Prot. Peptide Sci. 18, 1019–1034. doi: 10.2174/1389203717666160813164955, PMID: 27526928

[ref78] Qutob D. Patrick Chapman B. Gijzen M. (2013). Transgenerational gene silencing causes gain of virulence in a plant pathogen. Nat. Commun. 4:1349. doi: 10.1038/ncomms2354, PMID: 23322037 PMC3562452

[ref79] Raffaele S. Kamoun S. (2012). Genome evolution in filamentous plant pathogens: why bigger can be better. Nat. Rev. Microbiol. 10, 417–430. doi: 10.1038/nrmicro2790, PMID: 22565130

[ref80] Raman V. Simon S. A. Romag A. Demirci F. Mathioni S. M. Zhai J.. (2013). Physiological stressors and invasive plant infections alter the small RNA transcriptome of the rice blast fungus, *Magnaporthe oryzae*. BMC Genomics 14, 1–18. doi: 10.1186/1471-2164-14-326 23663523 PMC3658920

[ref81] Rao M. R. S. (2017). Long Non Coding RNA Biology. Cham: Springer.

[ref82] Reik W. Lewis A. (2005). Co-evolution of X-chromosome inactivation and imprinting in mammals. Nat. Rev. Genet. 6, 403–410. doi: 10.1038/nrg1602, PMID: 15818385

[ref83] Rhoades N. Nguyen T.-S. Witz G. Cecere G. Hammond T. Mazur A. K.. (2021). Recombination-independent recognition of DNA homology for meiotic silencing in *Neurospora crassa*. Proc. Natl. Acad. Sci. 118:e2108664118. doi: 10.1073/pnas.2108664118, PMID: 34385329 PMC8379962

[ref84] Sadakierska-Chudy A. Filip M. (2015). A comprehensive view of the epigenetic landscape. Part II: histone post-translational modification, nucleosome level, and chromatin regulation by ncRNAs. Neurotox. Res. 27, 172–197. doi: 10.1007/s12640-014-9508-6, PMID: 25516120 PMC4300421

[ref85] Separovich R. J. Wilkins M. R. (2021). Ready, SET, go: post-translational regulation of the histone lysine methylation network in budding yeast. J. Biol. Chem. 297:100939. doi: 10.1016/j.jbc.2021.100939, PMID: 34224729 PMC8329514

[ref86] Sharma Ghimire P. Jin C. (2017). Genetics, molecular, and proteomics advances in filamentous fungi. Curr. Microbiol. 74, 1226–1236. doi: 10.1007/s00284-017-1308-9 28733909

[ref87] Shen L. Song C.-X. He C. Zhang Y. (2014). Mechanism and function of oxidative reversal of DNA and RNA methylation. Annu. Rev. Biochem. 83, 585–614. doi: 10.1146/annurev-biochem-060713-035513, PMID: 24905787 PMC4786441

[ref88] Shimada A. Dohke K. Sadaie M. Shinmyozu K. Nakayama J.-I. Urano T.. (2009). Phosphorylation of Swi6/HP1 regulates transcriptional gene silencing at heterochromatin. Genes Dev. 23, 18–23. doi: 10.1101/gad.1708009, PMID: 19136623 PMC2632169

[ref89] Shiu P. K. Raju N. B. Zickler D. Metzenberg R. L. (2001). Meiotic silencing by unpaired DNA. Cell 107, 905–916. doi: 10.1016/S0092-8674(01)00609-2 11779466

[ref90] Simon S. A. Meyers B. C. (2011). Small RNA-mediated epigenetic modifications in plants. Curr. Opin. Plant Biol. 14, 148–155. doi: 10.1016/j.pbi.2010.11.007, PMID: 21159545

[ref91] Sims R. J. Nishioka K. Reinberg D. (2003). Histone lysine methylation: a signature for chromatin function. Trends Genet. 19, 629–639. doi: 10.1016/j.tig.2003.09.007 14585615

[ref92] Skinner M. K. (2015). Environmental epigenetics and a unified theory of the molecular aspects of evolution: a neo-Lamarckian concept that facilitates neo-Darwinian evolution. Genome Biol. Evol. 7, 1296–1302. doi: 10.1093/gbe/evv073, PMID: 25917417 PMC4453068

[ref93] Soyer J. L. El Ghalid M. Glaser N. Ollivier B. Linglin J. Grandaubert J.. (2014). Epigenetic control of effector gene expression in the plant pathogenic fungus *Leptosphaeria maculans*. PLoS Genet. 10:e1004227. doi: 10.1371/journal.pgen.1004227, PMID: 24603691 PMC3945186

[ref94] Stergiopoulos I. de Wit P. J. (2009). Fungal effector proteins. Annu. Rev. Phytopathol. 47, 233–263. doi: 10.1146/annurev.phyto.112408.132637 19400631

[ref95] Svoboda P. (2020). Introduction to RNAi and miRNA pathways. Nakladatelství Karolinum. Praha: Karolinum Press.

[ref96] Tahir M. S. Tian L. (2021). HD2-type histone deacetylases: unique regulators of plant development and stress responses. Plant Cell Rep. 40, 1603–1615. doi: 10.1007/s00299-021-02688-3, PMID: 34041586

[ref97] Tamaru H. Selker E. U. (2001). A histone H3 methyltransferase controls DNA methylation in *Neurospora crassa*. Nature 414, 277–283. doi: 10.1038/35104508 11713521

[ref98] Thapa B. Shrestha A. (2020). Epigenetic mechanisms and its role in plant growth and development. J. Plant Biochem. Physiol. 8:255.

[ref99] Thon G. Hansen K. R. Altes S. P. Sidhu D. Singh G. Verhein-Hansen J.. (2005). The Clr7 and Clr8 directionality factors and the Pcu4 cullin mediate heterochromatin formation in the fission yeast *Schizosaccharomyces pombe*. Genetics 171, 1583–1595. doi: 10.1534/genetics.105.048298, PMID: 16157682 PMC1456086

[ref101] Turner B. M. (2009). Epigenetic responses to environmental change and their evolutionary implications. Philos. Trans. R. Soc. B Biol. Sci. 364, 3403–3418. doi: 10.1098/rstb.2009.0125, PMID: 19833651 PMC2781845

[ref102] Uysal F. Akkoyunlu G. Ozturk S. (2015). Dynamic expression of DNA methyltransferases (DNMTs) in oocytes and early embryos. Biochimie 116, 103–113. doi: 10.1016/j.biochi.2015.06.019, PMID: 26143007

[ref103] Vu B. V. Pham K. T. M. Nakayashiki H. (2013). Substrate-induced transcriptional activation of the MoCel7C cellulase gene is associated with methylation of histone H3 at lysine 4 in the rice blast fungus *Magnaporthe oryzae*. Appl. Environ. Microbiol. 79, 6823–6832. doi: 10.1128/AEM.02082-13, PMID: 23995923 PMC3811509

[ref104] Whisson S. C. Vetukuri R. R. Avrova A. O. Dixelius C. (2012). Can silencing of transposons contribute to variation in effector gene expression in *Phytophthora infestans*? Mob. Genet. Elem. 2, 110–114. doi: 10.4161/mge.20265, PMID: 22934246 PMC3429519

[ref105] Wu G. Zhou H. Zhang P. Wang X. Li W. Zhang W.. (2016). Polyketide production of pestaloficiols and macrodiolide ficiolides revealed by manipulations of epigenetic regulators in an endophytic fungus. Org. Lett. 18, 1832–1835. doi: 10.1021/acs.orglett.6b00562, PMID: 27015125

[ref106] Xu Y. Miao Y. Cai B. Yi Q. Tian X. Wang Q.. (2022a). A histone deacetylase inhibitor enhances rice immunity by derepressing the expression of defense-related genes. Front. Plant Sci. 13:1041095. doi: 10.3389/fpls.2022.1041095 36407628 PMC9667192

[ref107] Xu Y. Miao Y. Tian X. Wang Q. Hu Y. Luo Q. (2022b). Transcriptomic and epigenomic assessment reveals epigenetic regulation of WRKY genes in response to *Magnaporthe oryzae* infection in rice. Curr. Genomics 23, 182–194. doi: 10.2174/1389202923666220510195910, PMID: 36777006 PMC9878826

[ref108] Yu C. Qi J. Han H. Wang P. Liu C. (2023). Progress in pathogenesis research of *Ustilago maydis,* and the metabolites involved along with their biosynthesis. Mol. Plant Pathol. 24, 495–509. doi: 10.1111/mpp.13307, PMID: 36808861 PMC10098057

[ref1003] Zanetti M. E. Blanco F. Ferrari M. Ariel F. Benoit M. Niebel A.. (2024). Epigenetic control during root development and symbiosis. Plant Physiol. 196, 697–710. 38865442 10.1093/plphys/kiae333

[ref109] Zhang Q. Chen L. Yu X. Liu H. Akhberdi O. Pan J.. (2016). AB-type histone acetyltransferase Hat1 regulates secondary metabolism, conidiation, and cell wall integrity in the taxol-producing fungus *Pestalotiopsis microspora*. J. Basic Microbiol. 56, 1380–1391. doi: 10.1002/jobm.201600131, PMID: 27400176

[ref110] Zhang Y. Yu W. Lu Y. Wu Y. Ouyang Z. Tu Y.. (2024). Epigenetic regulation of fungal secondary metabolism. J. Fungi 10:648. doi: 10.3390/jof10090648, PMID: 39330408 PMC11433216

[ref111] Zhao Y. Garcia B. A. (2015). Comprehensive catalog of currently documented histone modifications. Cold Spring Harb. Perspect. Biol. 7:a025064. doi: 10.1101/cshperspect.a025064, PMID: 26330523 PMC4563710

[ref112] Zhao F. Sun C. Liu Z. Cabrera A. Escobar M. Huang S.. (2022). Multiplex base-editing enables combinatorial epigenetic regulation for genome mining of fungal natural products. J. Am. Chem. Soc. 145, 413–421. doi: 10.1021/jacs.2c10211, PMID: 36542862 PMC10162584

